# Organizational culture and climate as moderators of enhanced outreach for persons with serious mental illness: results from a cluster-randomized trial of adaptive implementation strategies

**DOI:** 10.1186/s13012-018-0787-9

**Published:** 2018-07-09

**Authors:** Shawna N. Smith, Daniel Almirall, Katherine Prenovost, David E. Goodrich, Kristen M. Abraham, Celeste Liebrecht, Amy M. Kilbourne

**Affiliations:** 1 Department of Psychiatry, University of Michigan Medical School, Ann Arbor, MI USA; 2 Department of Internal Medicine, Division of General Medicine, University of Michigan Medical School, Ann Arbor, MI USA; 3 Institute for Social Research and Department of Statistics, University of Michigan, Ann Arbor, MI USA; 4 VA Center for Clinical Management Research, VA Ann Arbor Healthcare System, Ann Arbor, MI USA; 5 Department of Psychology, University of Detroit Mercy, Detroit, MI USA; 6 Health Services Research and Development, Veterans Health Administration, US Department of Veterans, Washington DC, USA

**Keywords:** Serious mental illness, Organizational culture, Organizational climate, Implementation science, Facilitation

## Abstract

**Background:**

Organizational culture and climate are considered key factors in implementation efforts but have not been examined as moderators of implementation strategy comparative effectiveness. We investigated organizational culture and climate as moderators of comparative effectiveness of two sequences of implementation strategies (Immediate vs. Delayed Enhanced Replicating Effective Programs [REP]) combining Standard REP and REP enhanced with facilitation on implementation of an outreach program for Veterans with serious mental illness lost to care at Veterans Health Administration (VA) facilities nationwide.

**Methods:**

This study is a secondary analysis of the cluster-randomized Re-Engage implementation trial that assigned 3075 patients at 89 VA facilities to either the Immediate or Delayed Enhanced REP sequences. We hypothesized that sites with stronger entrepreneurial culture, task, or relational climate would benefit more from Enhanced REP than Standard REP. Veteran- and site-level data from the Re-Engage trial were combined with site-aggregated measures of entrepreneurial culture and task and relational climate from the 2012 VA All Employee Survey. Longitudinal mixed-effects logistic models examined whether the comparative effectiveness of the Immediate vs. Delayed Enhanced REP sequences were moderated by culture or climate measures at 6 and 12 months post-randomization. Three Veteran-level outcomes related to the engagement with the VA system were assessed: updated documentation, attempted contact by coordinator, and completed contact.

**Results:**

For updated documentation and attempted contact, Veterans at sites with higher entrepreneurial culture and task climate scores benefitted more from Enhanced REP compared to Standard REP than Veterans at sites with lower scores. Few culture or climate moderation effects were detected for the comparative effectiveness of the full sequences of implementation strategies.

**Conclusions:**

Implementation strategy effectiveness is highly intertwined with contextual factors, and implementation practitioners may use knowledge of contextual moderation to tailor strategy deployment. We found that facilitation strategies provided with Enhanced REP were more effective at improving uptake of a mental health outreach program at sites with stronger entrepreneurial culture and task climate; Veterans at sites with lower levels of these measures saw more similar improvement under Standard and Enhanced REP. Within resource-constrained systems, practitioners may choose to target more intensive implementation strategies to sites that will most benefit from them.

**Trial registration:**

ISRCTN: ISRCTN21059161. Date registered: April 11, 2013.

**Electronic supplementary material:**

The online version of this article (10.1186/s13012-018-0787-9) contains supplementary material, which is available to authorized users.

## Background

For patients with serious mental illness (SMI), such as bipolar and schizophrenia spectrum disorders, continuity of care with healthcare providers can be the key to improving vulnerability to mortality and morbidity from preventable health conditions. Patients with SMI experience disproportionate rates of morbidity and early mortality from conditions like cancer and cardiovascular disease [1–3]. Gaps in the continuity of care and barriers in access to care access exacerbate this vulnerability [4, 5]. Outreach programs can improve continuity of care by improving ongoing assessment of patient needs and ensuring care outreach to patients with SMI. However, like other evidence-based practices, outreach programs often face barriers to implementation at both the provider- and system-levels, including complexities of coordinating care across multiple providers for patients with SMI [6], competing demands on provider time, and lack of leadership support [7–9].

Development and deployment of implementation strategies, or operationalized techniques based on underlying theories or frameworks designed to improve uptake of effective programs across diverse health care settings [10], can help sites address barriers by providing tools that promote program adoption and offer potential solutions. A large, growing toolbox of implementation strategies exists, with strategies spanning the range of effort, intensity, and cost [11–13]. This toolkit affords implementation scientists the ability to tailor the provision of implementation strategies to different healthcare site needs or capabilities to effectively and cost-efficiently encourage implementation of mental health interventions.

To date, however, few studies have examined *whether or how* the comparative effectiveness of different implementation strategies is moderated by organizational characteristics [14, 15]. For example, are more resource-intensive strategies more effective at sites lacking organizational support or capability, or are there organizational precursors that moderate the effectiveness of more intensive strategies? This toolkit of strategies also affords opportunities for providing *sequences* of implementation strategies that could be adapted to changing site needs [16–19]. Understanding how organizational characteristics moderate implementation strategy effectiveness, or differences in implementation strategy effectiveness across organizational settings, can help practitioners to better target specific implementation strategies (or sequences of strategies) to capitalize on or address different site needs or strengths and improve implementation efforts.

In 2012, the Veterans Health Administration (VA) issued a national policy directive requiring all eligible VA sites nationwide to implement Re-Engage, an outreach program for Veterans with an SMI diagnosis who had been lost to VA care. As part of the nationwide rollout of this project, the Re-Engage implementation trial [16] randomized sites to one of two sequences of implementation strategies that combined two implementation strategies, Standard and Enhanced Replicating Effective Programs (REP) (Table 1). REP is an implementation strategy based on the Centers for Disease Control’s Research-to-Practice Framework [20–23] and derived from Rogers’ diffusion model [24] and Social Learning Theory [25]. The Standard REP package consists of three components: user-friendly treatment program “packaging,” or translated treatment materials designed for easy dissemination; structured training for providers; and technical assistance for providers focused on the technical elements of implementation [26, 27]. The Standard REP strategy has proven effective at promoting treatment adoption and implementation [21, 22] but was anticipated to be inadequate for implementing a complex, multi-faceted program like Re-Engage across a diverse set of VA sites. In particular, Standard REP seemed unlikely to address common provider and system barriers to implementation, including lack of leadership support, multiple demands on provider time, and need to coordinate care across different clinics (e.g., medical and mental health) [16].

**Table 1:** Components of Standard vs. Enhanced Replicating Effective Programs implementation strategies

Component	Description	Standard REP	Enhanced REP
Package	Implementation manual disseminated with specific guidance on program components	x	x
Training	Program website coupled with virtual provider training and technical support via phone	x	x
Technical assistance	Monthly assessments of program process from each site with biweekly conference calls to address program deployment questions	x	x
External facilitation			
Gather information	Facilitators obtain background from different sources on site and potential barriers/facilitators		x
Ongoing partnership support	Facilitators hold regular (weekly) calls with site Re-Engage providers and also consult with additional regional leaders		x
Garner regional and local support	Facilitators provide ongoing information on Re-Engage progress to regional leadership		x
Identify barriers and facilitators	Facilitators and mental health providers continue regular (monthly) calls so facilitators can provide guidance on program implementation and address barriers to implementation		x
Collaboratively develop action plans	Working with site mental health provider, facilitators co-develop recommendations to mitigate barriers to Re-Engage and enhance program implementation based on providers’ strengths		x
Feedback and link to available resources	Facilitators provide a summary of progress and recommendations to providers, hand-off to additional resources, and summarize progress with leadership		x

From Kilbourne et al. 2013 [16]

The Enhanced REP implementation strategy augments Standard REP with ongoing external facilitation to help with the development of strategic skills necessary for addressing barriers related to providers or leaders [16, 22]. Facilitation, based on the integrated Promoting Action on Research Implementation in Health Services (i-PARiHS) framework [28–31], is a deliberate, interactive process of problem-solving and support that occurs in the context of a recognized need for assistance [32]. During facilitation, program experts engage with frontline providers to identify and problem-solve barriers to implementation [33–35]. For Re-Engage, facilitation was done through one-on-one phone consultations between providers and one of three study facilitators. In a prior study, Enhanced REP was found to improve fidelity to an evidence-based mental health collaborative care model relative to Standard REP in community-based practices [36].

### Organizational culture and climate as moderators of Enhanced vs. Standard REP

Study results [19, 37] found that Enhanced REP improved Re-Engage uptake more than Standard REP and was most effective with immediate, rather than delayed, provision. In an effort to better inform which VA sites most benefitted from different strategies, this paper examined whether these results were moderated by three previously validated measures of organizational culture or climate.

*Organizational culture* is defined as the shared values, norms, and expectations governing organizational behavior [38, 39]. As organizational culture is thought to be generally stable and difficult to change [40, 41], it can establish organizational priorities and impact individual behaviors and work processes in subconscious ways [42]. *Organizational climate* is defined as the “shared meaning organizational members attach to events, policies, practices, and procedures and […] the behaviors they see being rewarded, supported, and expected” [43, 44]. While measures of culture capture broader organizational values, norms, and priorities, measures of climate focus on employee perceptions of these norms as manifested through specific channels, such as rewards and stated priorities [45–47]. We explore both climate and culture as it is unclear whether broader cultural norms or the operationalization of those norms via climate [47] might better moderate implementation strategy effectiveness and be useful in tailoring implementation strategy provision.

While numerous measures of culture and climate exist [45–47], this paper uses three validated measures available for all VA sites: entrepreneurial culture and task and relational climate. *Entrepreneurial culture* is one of the four dominant cultures identified in the Competing Values Framework (CVF), which looks at organizational culture through two dimensions of competing values: centralization vs. de-centralization and internal vs. external focus [48]. Entrepreneurial organizations are de-centralized with an external focus; they are thought to value adoption of new practices, adaptation to external stimuli, and flexibility [42, 49]. We opted to focus on entrepreneurial culture in lieu of other CVF dimensions for theoretical and measurement-related reasons [42]. For climate, we examined *task* and *relational climate* measures [50–52]. *Task climate* refers to employee perceptions of managerial focus on improvement and achievement. *Relational climate* refers to managerial focus on support and respect [50]. Note that while task and relational climate are often highly correlated, collapsing the dimensions has been shown to obscure key distinctions between constructs [52].

Prior literature provides a foundation for these specific measures as potential moderators of REP enhanced with facilitation vs. Standard REP. While questions remain regarding how and why facilitation is effective in supporting implementation efforts [32, 53], successful facilitation efforts have been conceptualized as “realizing the latent learning capacity of organizations” [53]. While determinants of organizational latent learning capacity are not specified, the i-PARiHS framework identifies contextual factors—notably leadership support, receptivity to change, and cultural norms [29–31]—as key elements of successful implementation efforts. To the extent that measures of organizational culture/climate capture these contextual factors, and these contextual factors explain differences in a latent learning capacity, then, we would expect that differences in the effectiveness of facilitation might be explained by differences in organizational culture or climate.

Empirical research has also shown that successful facilitation efforts involve mobilizing existing knowledge bases and motivation for change [32, 54]; leveraging and improving site communications, relationships, and team building [55]; and aligning initiative goal setting and accomplishments with site priorities [55]. Such findings further buoy our consideration of entrepreneurial culture and task and relational climate as potential moderators of Enhanced REP effectiveness.

#### Entrepreneurial culture

Organizational values of flexibility and innovation have been connected to more successful clinical innovation implementation [56–58] and quality improvement [56, 59–65]. Successful facilitation efforts require providers to operate in contexts conducive to carrying out solutions offered by facilitators, with strong leadership engagement [66, 67], motivation for implementation [32, 68], and team functioning, including across professional boundaries [32, 68–70], and features aligning with organizational values of flexibility and innovation. Sites with high entrepreneurial culture may also show more support for “early adoption” of new initiatives, which may ensure that providers working with facilitators are more receptive to facilitator advice and/or that leadership is more supportive of implementation efforts and motivated to address implementation barriers. Providers at more entrepreneurial sites may also leverage site flexibility and innovation in acting on facilitator advice. For example, pilot Re-Engage efforts reported that one common implementation barrier was finding current residential information to update the Veteran’s status from sources beyond VA patient health records. Facilitators might recommend overcoming this barrier by examining novel sources of information (e.g., obituaries, websites, telephone-based information services) for updating patient information [71]. Providers at sites that value creativity and innovation may be more likely to follow through with such strategies.

#### Task climate

Sites with strong task climate feature performance-aligned goals, an emphasis on embracing change to improve work processes, and concrete notions of goal setting and organizational responsiveness [50, 72]. Much of Re-Engage implementation was highly task-oriented and involved reworking workflow processes to find, contact, and re-engage patients lost to care. Providers at sites where priorities aligned with efforts to enact such processes may have gained more from facilitation. Facilitators may also have more success in working with providers at strong task climate sites because these providers may be accustomed to carrying out tasks in a systematic manner—e.g., tracking implementation progress; setting concrete, near-term goals; and responding to feedback [55].

#### Relational climate

Relational climate has also been linked to better patient ratings of care quality [73] and higher quality chronic care provision [50, 51]. Sites with stronger relational climate tend to support trust and improved collaboration among staff, which may also contribute to improved facilitation effectiveness. Pilot Re-Engage efforts found that coordinating across providers for scheduling Veteran appointments was a common barrier to Re-Engage implementation. Facilitators often suggested overcoming these barriers by increasing outreach to other care providers or communicating with site leadership about VA policies and priorities [71]. Both of these solutions are likely to be more easily implemented at sites where there is an emphasis on teamwork and collaboration.

### Hypotheses

From prior theory, we hypothesized that among sites that were initially non-responsive to a low-level implementation strategy (Table 2):

- *Hypothesis 1*: Veterans at sites with higher entrepreneurial culture scores would *benefit more* from Enhanced REP than those at sites with lower entrepreneurial culture scores.
- *Hypothesis 2*: Veterans at sites with higher task climate scores would *benefit more* from Enhanced REP than those at sites with lower task climate scores.
- *Hypothesis 3*: Veterans at sites with higher relational climate scores would *benefit more* from Enhanced REP than those at sites with lower relational climate scores.

**Table 2:** Culture and climate hypotheses by treatment arm and 6-month phase

	Entrepreneurial culture Hypothesis 1	Task climate Hypothesis 2	Relational climate Hypothesis 3
First 6 months			
Immediate Enhanced REP (all sites get Enhanced REP)	More effective for sites with high scores	More effective for sites with high scores	More effective for sites with high scores
Delayed Enhanced REP (all sites get Standard REP)	–	–	–
Second 6 months			
Immediate Enhanced REP (all sites get Standard REP)	–	–	–
Delayed Enhanced REP (continued non-responders get Enhanced REP, responders receive Standard REP)	More effective for sites with high scores	More effective for sites with high scores	More effective for sites with high scores

*REP* Replicating Effective Programs

Three patient-level care management outcomes were examined: updated patient documentation, attempted patient contact, and completed patient contact.

## Methods

This study was a secondary analysis of a two-arm cluster-randomized controlled implementation trial comparing two sequences of implementation strategies to enhance uptake of the VA Re-Engage Program. The full study protocol has been published elsewhere [16]. The study was reviewed and approved by the local Institutional Review Board and registered as a Clinical Trial (Current Controlled Trials ISRCTN21059161). A summary of the Re-Engage program and implementation trial is presented below along with descriptions of new analyses presented here.

### The Re-Engage policy directive and program

Re-Engage, described in full elsewhere [16, 19, 37, 71, 74–76], is an outreach program comprising an assessment of the patient’s current status and outreach for Veterans with SMI. Re-Engage is intended to remedy disparities in mortality and morbidity brought about by discontinuous care [77] by “re-engaging” in VA care Veterans with SMI who had been lost to VA care for 1 year or more. Early demonstration projects showed substantial and significant reductions in mortality for Veterans with SMI who returned to care, with Veterans returned to care showing an adjusted mortality rate of 0.5% compared to 3.9% among Veterans who did not return to care [75, 78]. In response to these findings, VA leadership implemented Re-Engage as part of standard care through a national mandate.

The Re-Engage policy directive took effect on March 1, 2012. Providers at all VA facilities were provided with a list of Veterans with SMI lost to care (no VA outpatient visits and no inpatient stays of more than 2 days for at least 12 months) [16, 76]. VA facilities with at least one Veteran with SMI documented as lost to care were required to implement Re-Engage.

As part of Re-Engage, sites were to designate a mental health provider (Local Recovery Coordinator, LRC) to implement Re-Engage [76]. Following a comprehensive chart review, the provider was to attempt to contact the Veteran, if appropriate, and, if successfully contacted, assess clinical need and facilitate a return to VA care by helping the Veteran to schedule an appointment [16, 76, 79]. Subsequently, LRCs were to document each listed Veteran’s updated disposition and results of outreach efforts in a database maintained by the VA Serious Mental Illness Treatment Resource and Evaluation Center (SMITREC; a program evaluation center formerly located in the VA Office of Mental Health Operations, now in the Office of Mental Health and Suicide Prevention) for purposes of program monitoring and evaluation.

### Re-Engage cluster-randomized implementation trial

Full eligibility criteria for the Re-Engage implementation trial has been detailed elsewhere [16, 19]. The target unit for the implementation interventions was the VA site.

**Fig. 1:**
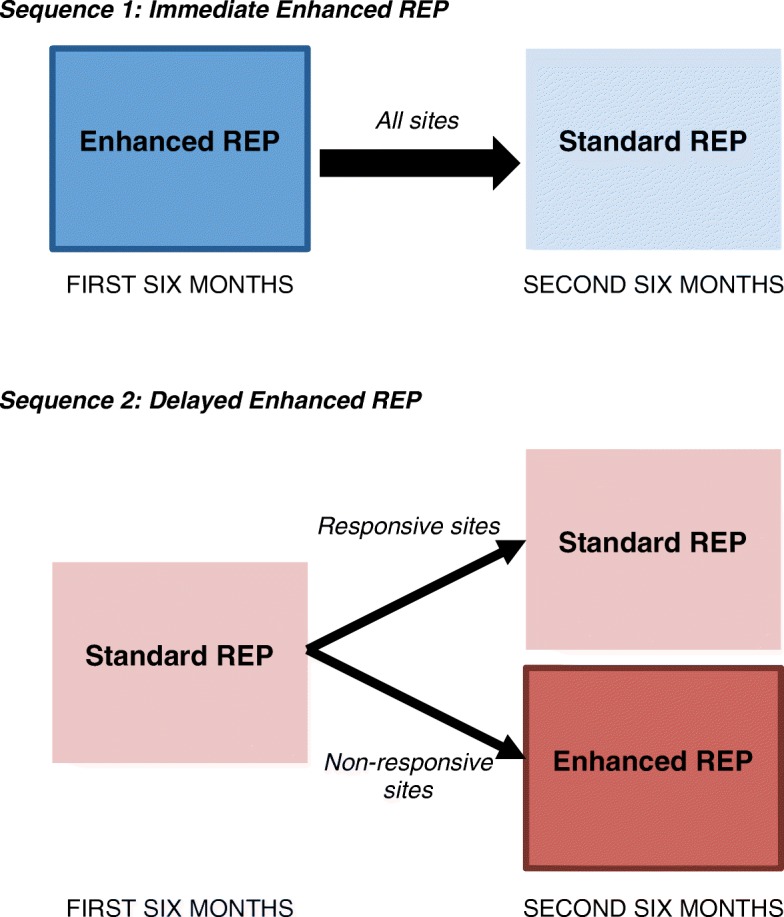
Two sequences of implementation strategies. Note: REP = Replicating Effective Programs. For sequence 2, responsive sites were those where 80% of listed patients had updated documentation

Eighty-nine sites (*n* = 3075 Veterans) were considered non-responsive to Standard REP, defined as having updated documentation for less than 80% of listed Veterans with SMI after 6 months. These sites were then randomized (at the regional network (VISN) level) to one of two sequences of implementation strategies (Fig. 1). *Immediate Enhanced REP* (*N* = 40 sites; *n* = 1543 Veterans) provided Enhanced REP for 6 months and then stepped down to Standard REP for a further 6 months. *Delayed Enhanced REP* (*N* = 49 sites; *n* = 1532 Veterans) was an adaptive implementation strategy that continued to provide Standard REP for a further 6 months and then evaluated response again. Continued non-responsive sites (*N* = 35) then received Enhanced REP for the second 6 months while responsive sites (*N* = 14) continued to receive Standard REP. All study outcomes were evaluated at 6 and 12 months.

### Organizational culture and climate measures

Organizational culture and climate measures came from the 2012 VA All Employee Survey (AES), a national survey of employees focused on organizational culture and climate distributed anonymously on a yearly basis. This survey and measures have been described elsewhere [80]. The 2012 AES was fielded (and all moderators measured) prior to any trial randomization (April 23 to May 14, 2012), yielding 173,413 responses nationwide (63.4% response rate). Our analyses employed site-aggregated AES data provided through the VA Employee Survey Portal, part of the VA Service Support Center, for all 89 sites included in the Re-Engage trial. Availability of only site-aggregated data precluded us from justifying our level of aggregation via, e.g., measures of within-site agreement. Several prior studies, however, have demonstrated suitably large within-site agreement of climate and culture measures to justify aggregation [50, 81].

Entrepreneurial culture measures came from the AES’ “culture” battery of questions, which included four items for each CVF dimension. The “Organizational Assessment Inventory” included three items related to each task and relational climate (Table 3). All items were measured on a 5-point Likert scale ranging from strongly disagree (1) to strongly agree (5). Confirmatory factor analyses (CFA), reported elsewhere [80], were used to validate the culture and climate measures for use with the 2012 VA AES. To account for differences in item weights [80], factor scores were created from CFA factor loadings and standardized to a mean of 0 and unit variance, with higher scores indicating higher levels of the particular culture/climate.

**Table 3:** VA All Employee Survey items comprising culture and climate measures

	Entrepreneurial culture	Task climate	Relational climate
1. My facility is a very dynamic and entrepreneurial place. People are willing to stick their necks out and take risks.	x		
2. Managers in my facility are risk-takers. They encourage employees to take risks and be innovative.	x		
3. The glue that holds my facility together is the commitment to innovation and development. There is an emphasis on being first.	x		
4. My facility emphasizes growth and acquiring new resources. Readiness to meet new challenges is important.	x		
7. The glue that holds my facility together is the formal rules and policies. People feel that following the rules is important.	x		
9. New practices and ways of doing business are encouraged in my work.		x	
10. Managers set challenging and yet attainable performance goals for my work group.		x	
11. Employees in my work group are involved in improving the quality of products, services, and work processes.		x	
12. Disputes or conflicts are resolved fairly in my work group.			x
13. A spirit of cooperation and teamwork exists in my work group.			x
14. Differences among individuals are respected and valued in my work group.			x

Items and loadings for each factor were determined through confirmatory factor analysis detailed in Smith et al. [80]. *VA* Veterans Health Administration

### Dependent variables: implementation outcomes

Three patient-level implementation outcomes, described elsewhere [16, 19, 37], were assessed at 6 and 12 months post-randomization: updated documentation, attempted contact, and completed contact. Updated documentation, the primary implementation outcome for the Re-Engage implementation trial [16, 19, 37], refers to whether LRCs updated Veteran records to reflect their current clinical and social disposition, based on current VA medical record entries, or other information related to the patient’s location or contact information. This patient-level variable was considered the primary implementation outcome as it best captures site-level implementation efforts and is not affected by, e.g., differences in patient populations that might affect the difficulty of attempting or completing patient contact. It is a clear indicator of whether the program is being carried out. Attempted contact was recorded for a Veteran if the LRC attempted to contact them by phone, through next of kin, or by mail. Completed contact was documented if the Veteran was successfully reached and need for service was ascertained. All measures were collected as part of standard program monitoring via a VA web-based registry using the Inquisite software package.

### Analysis strategy

Table 2 summarizes hypotheses 1–3 as they relate to each 6-month period for each study arm. Note that these hypotheses refer specifically to moderation effects on Enhanced REP vs. Standard REP during either the first or second 6-month periods, but not to the *sequence* of Immediate vs. Delayed Enhanced REP. Analyses examining culture and climate measures as they moderate the comparative effectiveness of full sequences on 12-month outcomes were considered exploratory.

Main effects of the Re-Engage trial after 12 months have been reported elsewhere [19]. Our analyses used three-level longitudinal mixed-effects binary logistic models to compare the effects of Immediate vs. Delayed Enhanced REP over time as moderated by each measure of culture/climate on outcomes after 12 months. All regressions included an intercept, main effect for treatment (Immediate Enhanced REP vs. Delayed Enhanced REP arm) at baseline, 6 and 12 months; main effect for the culture/climate factor score at baseline, 6 and 12 months; and an interaction between treatment arm and culture/climate at baseline, 6 and 12 months. The significance of the interaction term between treatment and culture/climate was used to evaluate our hypotheses for whether treatment effects were moderated by organizational culture/climate. Models also included independent random intercepts for the site and VISN with variances assumed to be independent and normally distributed. Patient-level random effects were also explored but were largely co-varying with site-level random effects and therefore not included. Following from prior work [37, 78], we adjusted all models for pre-randomization Veteran age, gender, number of medical comorbidities, marital status, VA service connection, homelessness, diagnosis of schizophrenia or related disorder, and whether the last VA visit was an inpatient visit, and pre-randomization site-level measures of facility size (number of unique patients in FY 2012), outpatient clinic vs. VA medical center, and total number of Veterans with SMI at the site identified as lost to follow-up care. All analyses were performed in Stata version 15.1 using *melogit* commands.

## Results

Overall, 3075 Veterans from 89 VA sites were included in the study. Table 4 reports descriptive statistics for patient- and site-level covariates, culture/climate measures, and outcomes after 6 and 12 months by arm. Outcomes were recorded at three time points, for a total of 9225 data points. As previously reported [19], Veterans in the Immediate Enhanced REP arm were slightly more likely to be Black and be diagnosed with schizophrenia. Sites in the Immediate Enhanced REP arm also had significantly higher task and relational climate scores, but no significant differences were found with respect to entrepreneurial culture.

**Table 4:** Characteristics of study sites and Veterans, by treatment arm (*N*, %)

	Adaptive intervention #1: Immediate Enhanced REP	Adaptive intervention #2: Delayed Enhanced REP
Site characteristics (*N* = 89)	*N* = 40 sites	*N* = 49 sites
Outpatient care only?	4 (10.0)	6 (12.2)
Mean total # of patients in FY12 (SD)	41,427 (18,216)	38,865 (21,770)
Mean number of Veterans diagnosed with SMI lost to care on site list (SD)	17 (6)	17 (7)
Culture and climate measures		
Mean entrepreneurial culture (SD)	− 0.06 (1.01)	− 0.17 (1.07)
Mean task climate (SD)	0.22 (1.03)	− 0.28 (0.94)*
Mean relational climate (SD)	0.19 (1.03)	− 0.28 (0.97)*
Veteran characteristics (*n* = 3075)	*n* = 1543 Veterans	*n* = 1532 Veterans
Male	1405 (91.1)	1377 (89.9)
Black	329 (21.3)	235 (15.3)*
Married	405 (26.3)	410 (26.8)
Service connected	424 (27.5)	437 (28.5)
Homeless	229 (14.8)	194 (12.7)
Schizophrenia diagnosis	683 (44.3)	595 (38.8)*
Last site visit inpatient?	69 (4.5)	69 (4.5)
Veteran outcomes (*N* = 3075)	*N* = 1543 Veterans	*N* = 1532 Veterans
Updated documentation		
First 6 months	605 (39.2)	262 (17.1)*
Second 6 months	243 (15.7)	351 (22.9)*
All 12 months	848 (55.0)	613 (40.0)*
Attempted contact		
First 6 months	479 (31.0)	207 (13.5)*
Second 6 months	215 (13.9)	284 (18.5)*
All 12 months	694 (45.0)	491 (32.1)*
Completed contact		
First 6 months	121 (7.8)	57 (3.7)*
Second 6 months	77 (5.0)	85 (5.5)
All 12 months	198 (12.8)	142 (9.3)*

Immediate Enhanced REP refers to the adaptive intervention that starts with Enhanced REP for 6 months then steps all sites down to Standard REP for the second 6 months, while Delayed Enhanced REP refers to the adaptive intervention that starts with Standard REP for 6 months and then provides Enhanced REP for the second 6 months for sites that were still non-responsive

*P value of < 0.05 based on Rao-Scott chi-square test for categorical variables or two-tailed *t* test for continuous variables. *REP * Replicating Effective Programs *SD* standard deviation *SMI* Serious mental illness

Table 5 shows factor change coefficients and 95% confidence intervals (CI) from the three-level mixed-effects binary logit models for main treatment effects and interaction between treatment effect and culture/climate measures at 6 and 12 months (relative to baseline) for each outcome, by culture/climate measure of interest. Full model results are presented in Additional file 1. Factor change coefficients can be interpreted as the change in the odds of observing the dependent variable for a given change in *x*. As such, the main treatment effect can be interpreted as the difference in the odds of the dependent variable in the Immediate vs. Delayed Enhanced arm for each time period, while the interaction term indicates how much this difference in the odds across treatment arms varied by culture/climate score. For example, a positive interaction term for task climate indicates sites with higher task climate saw larger differences in the odds of the dependent variable under Immediate vs. Delayed Enhanced REP than sites with lower task climate scores.

**Table 5:** Patient-level factor change coefficients and 95% confidence intervals for effects of implementation strategy and culture and climate measures, by the outcome, for 6-month intervals and 12 months cumulative post-randomization (*N* = 9225)

	Entrepreneurial culture	Task climate	Relational climate
Updated documentation			
Immediate vs. Delayed Enhanced REP at 6 months (vs. baseline)	8.24 (5.74, 11.83)*	9.69 (6.58, 14.27)*	10.10 (6.83, 14.95)*
Immediate vs. Delayed Enhanced REP at 12 months (vs. baseline)	5.91 (3.99, 8.76)*	7.05 (4.63, 10.73)*	7.72 (5.04, 11.84)*
Immediate Enhanced REP x culture/climate score at 6 months (vs. baseline)	2.81 (1.98, 3.98)*	3.05 (2.05, 4.55)*	2.56 (1.72, 3.81)*
Immediate Enhanced REP x culture/climate score at 12 months (vs. baseline)	2.05 (1.41, 2.97)*	2.00 (1.30, 3.08)*	1.94 (1.27, 2.98)*
BIC statistic	7241.7	7271.8	7727.7
Attempted contact			
Immediate vs. Delayed Enhanced REP at 6 months (vs. baseline)	3.38 (2.42, 4.74)*	3.73 (2.64, 5.27)*	3.72 (2.64, 5.25)*
Immediate vs. Delayed Enhanced REP at 12 months (vs. baseline)	2.35 (1.67, 3.31)*	2.52 (1.76, 3.59)*	2.58 (1.81, 3.68)*
Immediate Enhanced REP x culture/climate score at 6 months (vs. baseline)	1.59 (1.12, 2.25)*	1.65 (1.14, 2.38)*	1.27 (0.88, 1.84)
Immediate Enhanced REP x culture/climate score at 12 months (vs. baseline)	0.99 (0.69, 1.41)	0.98 (0.67, 1.43)	0.79 (0.55, 1.16)
BIC statistic	7511.1	7531.0	7536.5
Completed contact			
Immediate vs. Delayed Enhanced REP at 6 months (vs. baseline)	1.59 (0.96, 2.63)	1.64 (0.97, 2.79)	1.66 (0.98, 2.78)
Immediate vs. Delayed Enhanced REP at 12 months (vs. baseline)	1.22 (0.76, 1.98)	1.15 (0.69, 1.90)	1.18 (0.72, 1.94)
Immediate Enhanced REP x culture/climate score at 6 months (vs. baseline)	1.07 (0.65, 1.77)	1.29 (0.74, 2.23)	1.01 (0.59, 1.75)
Immediate Enhanced REP x culture/climate score at 12 months (vs. baseline)	0.92 (0.58, 1.48)	1.11 (0.66, 1.88)	0.96 (0.57, 1.63)
BIC statistic	4873.4	4877.9	4880.8

*P value of <0.05. Factor change coefficients represent the change in the odds of observing the dependent variable for a given change in *x*. Immediate Enhanced REP refers to the adaptive intervention that starts with Enhanced REP for 6 months then steps all sites down to Standard REP for the second 6 months, while Delayed Enhanced REP refers to the adaptive intervention that starts with Standard REP for 6 months and then provides Enhanced REP for the second 6 months for sites that were still non-responsive. Interaction terms (e.g., Immediate Enhanced REP x culture/climate score at 6 months) indicate the difference in the treatment effect by culture/climate score. Full model adjusts for patient age, gender, number of medical comorbidities, marital status, VA service connection, homelessness, diagnosis of schizophrenia or related disorder, and site-level measures of facility size (number of unique patients in FY 2012), whether the site was an outpatient clinic or VA medical center, and total number of Veterans with SMI at the site identified as having been lost to follow-up care, and includes random intercepts for site and VISN. *REP* Replicating Effective Programs, *BIC* Bayesian information criterion

With respect to updated documentation, during the first 6 months after randomization, patients at sites with higher levels of culture/climate in the Immediate Enhanced REP treatment arm had the highest odds of updated documentation. This effect was attenuated between months 6 and 12, during which facilitation was ceased in the Immediate Enhanced REP arm and started in the Delayed Enhanced REP arm. Looking specifically at entrepreneurial culture, within the first 6 months, patients at all sites had higher odds of updated documentation under Immediate than Delayed Enhanced REP (OR = 8.24; CI 5.74, 11.83); further, patients at sites with higher levels of entrepreneurial culture benefitted more from Immediate Enhanced REP than Delayed Enhanced REP than those with lower levels of Entrepreneurial Culture (OR = 2.81, CI 1.98, 3.98). This moderation effect persisted over the full 12-month period but was less pronounced in later months (OR = 2.05, 95% CI 1.41, 2.97). This same pattern also held for the task and relational climate.

**Fig. 2:**
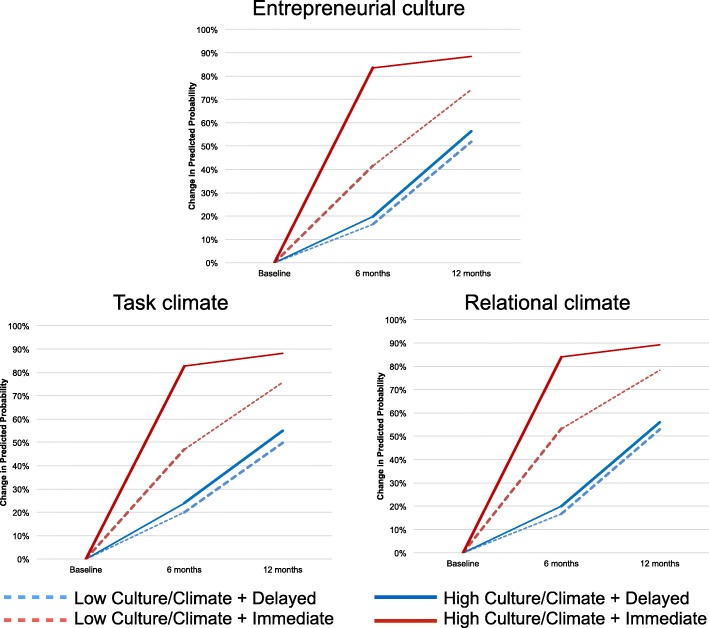
Predicted probability of updated documentation over time, by treatment arm and culture/climate score. Note: Predicted probabilities were computed for fixed effects only and were centered at the mean probability across treatment groups prior to randomization. Models examined the comparison of effects of Immediate Enhanced REP vs. Delayed Enhanced REP as moderated by culture and climate variables (see Fig. 1). High entrepreneurial culture probabilities were computed with entrepreneurial culture score at 1 standard deviation above the mean and low entrepreneurial culture probabilities at 1 standard deviation below the mean. Thicker lines denote the periods during which each some or all sites in each arm received Enhanced REP

Figure 2 illustrates this pattern of effects, showing the changes in the predicted probability of updated documentation during each 6-month period for sites with low culture/climate (one standard deviation below the mean; dashed lines) and high culture/climate (one standard deviation above the mean; solid lines) by treatment arm. All probabilities were computed for fixed effects with other variables held at their means and were centered at the mean probability across treatment groups prior to randomization. The plots show consistently that during the first 6 months, patients at high culture/climate sites in the Immediate Enhanced REP arm saw the biggest increases in the probability of updated documentation. Increases ranged from 83 (task) to 84 percentage points (relational) in the Immediate Enhanced REP arm compared to increases of 20 (entrepreneurial, task) or 24 percentage points (relational) for high culture/climate sites in the Delayed Enhanced REP arm. During the second 6 months, patients at sites in the Immediate Enhanced arm (both high and low culture/climate) saw smaller increases in their probability of updated documentation than those in the Delayed Enhanced REP arm—not unexpected given that most sites in the Delayed arm were receiving facilitation during the second 6 months. After 12 months, however, patients at sites with high culture/climate measures still had larger increases in their probabilities under Immediate Enhanced REP than Delayed (e.g., 88 vs. 56 percentage points for entrepreneurial culture), while patients at low culture/climate sites saw smaller differences across treatment arms (e.g., 74 vs. 52 percentage point for entrepreneurial culture).

**Fig. 3:**
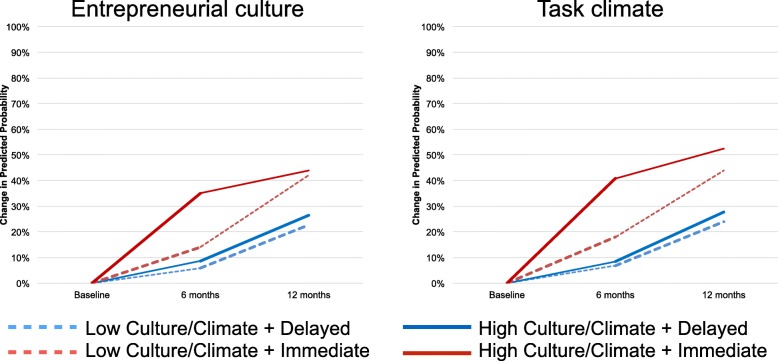
Predicted probability of attempted contact over time, by treatment arm and entrepreneurial culture score. Note: Predicted probabilities were computed for fixed effects only and were centered at the mean probability across treatment groups prior to randomization. Models examined the comparison of effects of Immediate Enhanced REP vs. Delayed Enhanced REP as moderated by culture and climate variables (see Fig. 1). High entrepreneurial culture probabilities were computed with entrepreneurial culture score at 1 standard deviation above the mean and low entrepreneurial culture probabilities at 1 standard deviation below the mean. Thicker lines denote the periods during which each some or all sites in each arm received Enhanced REP

Similar results were found for the attempted contact outcome (Table 5); however, moderation effects were only significant for entrepreneurial culture and task climate during the first 6 months; differences were largely attenuated by 12 months. Figure 3 illustrates changes in predicted probabilities for each 6-month period for entrepreneurial and task climate. For both measures, patients at sites with higher values saw larger increases in their probabilities of attempted contact under Immediate (entrepreneurial, 35 percentage points; task, 41 percentage points) than Delayed Enhanced REP (entrepreneurial, 9 percentage points; task, 8 percentage points) during the first 6 months than did those with low values on the culture/climate measures (entrepreneurial, 14 Immediate vs. 6 percentage points Delayed; task, 18 vs. 7 percentage points). These differences were attenuated during the second 6 months when sites with high culture/climate in the Delayed Enhanced REP arm saw larger increases than those in the Immediate Enhanced REP arm (entrepreneurial, 17 vs. 9 percentage points; task, 20 vs. 11 percentage points for task). Patients at sites with low culture/climate continued to see smaller increases across treatment arms (entrepreneurial, 28 Immediate vs. 17 percentage points Delayed; task: 26 Immediate vs. 17 percentage points Delayed).

No significant moderation effects were found for the completed contact outcome.

## Discussion

This paper used data from one of the first trials comparing two sequences of implementation strategies at VA sites nationwide. Specifically, we examined whether measures of organizational culture/climate moderated the comparative effectiveness of two implementation strategies, Enhanced vs. Standard REP, or two sequences of implementation strategies, Immediate vs. Delayed Enhanced REP, among sites not initially responsive after 6 months of Standard REP. We found that, for both updated documentation and attempted contact outcomes, Veterans at sites with higher culture/climate scores benefitted more from Enhanced vs. Standard REP, and from Immediate vs. Delayed Enhanced REP, than did Veterans at sites with lower culture/climate scores. These moderation effects were most pronounced during the first 6 months of the trial (when sites in the Immediate Enhanced REP arm received facilitation and sites in the Delayed arm did not) and were attenuated during the second 6 months when sites in the Delayed Enhanced REP arm received facilitation. After 12 months, sites with high entrepreneurial culture and task climate benefited significantly more from the Immediate vs. Delayed Enhanced REP than did sites with lower culture/climate measures for the updated documentation outcome; moderation effects for the attempted contact outcome were no longer significant after 12 months (although differences in predicted probabilities remained).

Returning to our hypotheses, we found support for hypotheses 1 and 2, with higher entrepreneurial culture and task climate scores associated with greater comparative effectiveness of Enhanced REP compared to Standard REP for two of three outcomes, and some support for hypothesis 3, with higher relational climate increasing the effectiveness of Enhanced REP on updated documentation, but not attempted contact.

The positive interactions between the culture/climate measures described here and the Enhanced REP implementation strategy were generally hypothesized based on prior theory and empirical evidence linking culture/climate to improved facilitation effectiveness. The findings suggest that, in fact, facilitation leverages conditions related to a site’s overarching norms of innovation or risk-taking or policies and procedures related to work processes and task attainment.

Additionally, however, our findings also show that, at least for our primary implementation outcome of updated patient documentation, after 12 months, patients at sites with high culture/climate measures who received Immediate Enhanced REP had significantly higher odds of updated documentation than patients at sites that had an opportunity to receive Enhanced REP 6 months later. One explanation may be that at sites with more amenable climates, facilitation provided early on was better able to leverage leadership attention to the Re-Engage mandate and/or interest in being an “early-ish” adopter. A second potential explanation is that in the Delayed Enhanced REP arm, sites with higher culture/climate were more likely to become responders and thus not receive facilitation when it was later offered. However, contrary to this supposition, we find that the entrepreneurial culture and task climate scores for sites in the Delayed Enhanced REP arm that were responders after 12 months were actually significantly lower than non-responding sites (*t* = 2.41, *P* = 0.02; *t* = 4.75, *P* < 0.01, respectively).

This does not explain, however, the lack of moderation effects with respect to completed contact. It may be that facilitation solutions that most leveraged the added benefits of entrepreneurial culture and/or task climate were simply further upstream from completed contact, or that, net the skills required for attempting contact, contact completion was a function of LRC skill or Veteran characteristics rather than organizational goals or priorities. The lower rate of completed contact also makes moderation effects more difficult to detect.

The findings of this study have several important implications for researchers and practitioners interested in implementing mental health outreach or management programs. First, our results suggest that measures of entrepreneurial culture and/or task climate may be useful for deciding how to triage more intensive implementation strategies. In particular, as sites with better entrepreneurial culture and/or task climate benefit more from Enhanced REP, practitioners looking to efficiently implement mental health programs may choose to provide Enhanced REP to only those sites. Just as implementation efforts gain nothing by providing more intensive implementation strategies to sites that would succeed under less intensive strategies, they also gain little in providing enhanced implementation strategies to sites that lack the capabilities to take advantage of the enhancements. As our results showed, sites with lower culture/climate scores fared nearly as well under Standard REP and Enhanced REP for both updated documentation and attempted contact outcomes during both the first and second 6 months. While Enhanced REP as operationalized for the Re-Engage study was not overly expensive (estimated at 7.5 h of facilitator time per site over 6 months [37]), large-scale implementation efforts may opt to reserve the added expenditure for sites that would benefit the most from the enhancement.

Such findings may seem counterintuitive. In an effort to offset site-level lack of resources with greater support, well-intentioned practitioners seeking to implement care management programs under constrained resources may be inclined to provide the most intensive implementation strategies to lower-resourced sites. Our results, however, suggest that such an approach could be misguided. Rather, practitioners might be better served by providing implementation support that works to build internal capacity, rather than support that leverages such capacity—e.g., additional trainings for site leadership and providers to align priorities or establish a priori channels of communication. As implementation science works to map implementation strategies to mechanisms of change [11, 12, 14], furthering research that better specifies which implementation strategies work best for different organizational contexts will help to strengthen these efforts. Such efforts to tailor strategy provision are likely to be more effective than efforts to change organizational culture or climate, which typically require timeframes beyond that of an evidence-based practice implementation intervention.

While most implementation scientists acknowledge the importance of organizational context on implementation efforts, significant gaps persist in our knowledge as to *whether and how* organizational factors enhance or impede quality improvement efforts [14, 15, 82]. Tying organizational characteristics to implementation strategy effectiveness also provides early clues to as to the mechanisms through which implementation strategies are most effective. In this case, the moderating effects of entrepreneurial culture and task climate on the effectiveness of REP enhanced with facilitation suggest that facilitation efforts work by leveraging existing resources in problem-solving efforts and/or alignment of duties with leadership priorities and that facilitation’s effectiveness may be limited by pre-existing deficiencies in organizational priorities or leadership support for innovation. Future work should build on these preliminary findings, perhaps through better collection of facilitation process data that better illustrates *how* facilitation leverages culture/climate, to better isolate the “active ingredient” of facilitation as an effective implementation strategy. More broadly, better task climate and entrepreneurial culture may also improve effectiveness of implementation efforts focused on aligning management and employee motivations to improve performance by combining top-down (results-driven, task climate-related) and bottom-up (provider-informed, entrepreneurial culture-related) efforts, e.g., evidence-based quality improvement [83].

This study has a number of limitations. Most notably, findings are specific to the VA setting and the Re-Engage outreach program and may not be generalizable beyond this particular system or program. Further, measures of entrepreneurial culture and task and relational climate examined were general measures (not related to Re-Engage or implementation efforts more generally). Program- or implementation-specific measures of climate, such as implementation leadership or climate measures [43, 84, 85], may have revealed different associations. Moreover, the measures evaluated were highly correlated, which make disentangling the true moderator(s) of interest more difficult. This collinearity is not surprising as, although these measures have been widely studied, they overlap to a strong degree both conceptually and operationally. Future efforts to tailor implementation strategy provision to organizational environment would be benefited by the development of “pragmatic” measures of organizational context that are conceptually unique, psychometrically sound, sensitive to change, and low burden to collect [86]. Available measures of culture and climate were also already aggregated at VA site level. This precluded both evaluation of within-site variation or exploration of more proximal levels of aggregation, e.g., the mental health unit responsible for implementing Re-Engage. Unit-specific measures of culture and climate may have revealed different associations with implementation strategy effectiveness. As a further limitation, leadership interest in rapid implementation of Re-Engage also limited the Enhanced REP intervention periods to 6 months [19]; given more time, moderating effects of entrepreneurial culture and task climate may have dissipated. Process data that would allow us to fully unpack the mechanisms behind the observed moderation effects was also not collected. Cost considerations and the nationwide scope of the study prevented inclusion of in-person Enhanced REP (i.e., internal facilitation). Future work should examine whether the effects of Enhanced REP including both internal and external facilitation [87] are similarly moderated by culture and/or climate, and whether more implementation process-specific measures moderate implementation strategy comparative effectiveness. Finally, the Re-Engage trial was not powered to detect moderation effects.

## Conclusion

Mental health care management and coordination programs hold potential for improving both physical and mental health outcomes for patients with SMI, but implementation efforts face numerous potential barriers. While a growing toolkit of implementation strategies exists to address these barriers, little is currently known as to how to tailor implementation strategy provision to site characteristics to ensure maximal uptake with minimal resources. Results of this national cluster-randomized implementation trial show that Veterans at sites with higher entrepreneurial culture and task climate saw larger increases in their odds of having updated documentation and an attempted contact under Enhanced REP including facilitation compared to Standard REP than did Veterans at sites with lower entrepreneurial culture or task climate. These results are instructive both for tailoring implementation strategy delivery and for illuminating potential mechanisms of effectiveness for the facilitation implementation strategy.

## Additional file

Additional file 1: **Table S1.** Full model results for updated documentation. **Table S2.** Full model results for attempted contact. **Table S3.** Full model results for completed contact. (PDF 316 kb)
