# Supplementary material for: Organizational culture and climate as moderators of enhanced outreach for persons with serious mental illness: results from a cluster-randomized trial of adaptive implementation strategies
Source: Implement Sci. 2018 Jul 9;13:93. doi: 10.1186/s13012-018-0787-9 (PMC6038326; doi:10.1186/s13012-018-0787-9)
Supplement: Supplementary file 1 — Table S1. Full model results for updated documentation. Table S2. Full model results for attempted contact. Table S3. Full model results for completed contact. (PDF 316 kb) [file 13012_2018_787_MOESM1_ESM.pdf]

Table S1: Updated documentation

Updated Documentation		Entrepreneurial Culture OR (.95 CI)	Hierarchical Culture OR (.95 CI)	Task Climate OR (.95 CI)	Relational Climate OR (.95 CI)
Immediate vs. Delayed EREP		0.20 (0.05,0.73)	0.16 (0.04,0.62)	0.15 (0.04,0.56)	0.16 (0.04,0.60)
Culture/Climate (standardized)		0.53 (0.26,1.08)	0.45 (0.21,0.96)	0.68 (0.34,1.33)	0.69 (0.35,1.34)
Immediate vs. Delayed EREP × Culture/Climate		1.00 (0.37,2.71)	1.94 (0.71,5.33)	0.78 (0.27,2.24)	0.94 (0.33,2.68)
Time					
	6 months (vs. 0 months)	4.18* (3.36,5.20)	4.18* (3.36,5.20)	4.33* (3.46,5.43)	4.25* (3.39,5.32)
	12 months (vs. 0 months)	19.74* (15.62,24.95)	20.14* (15.91,25.49)	19.67* (15.44,25.04)	19.23* (15.11,24.47)
Immediate vs. Delayed EREP × Time					
	6 months (vs. 0 months)	8.24* (5.74,11.83)	8.88* (6.14,12.83)	9.69* (6.58,14.27)	10.10* (6.83,14.95)
	12 months (vs. 0 months)	5.91* (3.99,8.76)	7.03* (4.68,10.56)	7.05* (4.63,10.73)	7.72* (5.04,11.84)
Culture/Climate × Time					
	6 months (vs. 0 months)	1.09 (0.87,1.37)	1.38 (1.10,1.73)	0.86 (0.69,1.08)	0.94 (0.74,1.18)
	12 months (vs. 0 months)	1.11 (0.88,1.40)	1.53* (1.22,1.92)	1.02 (0.81,1.28)	1.09 (0.86,1.37)
Immediate vs. Delayed EREP × Culture/Climate × Time					
	6 months (vs. 0 months)	2.81* (1.98,3.98)	2.26* (1.59,3.23)	3.05* (2.05,4.55)	2.56* (1.72,3.81)
	12 months (vs. 0 months)	2.05* (1.41,2.97)	2.20* (1.51,3.20)	2.00 (1.30,3.08)	1.94 (1.27,2.98)
Baseline Age (centered at 21 years)		1.00 (1.00,1.00)	1.00 (1.00,1.00)	1.00 (1.00,1.00)	1.00 (1.00,1.00)
Male (vs. Female)		1.11 (0.90,1.37)	1.11 (0.90,1.37)	1.11 (0.90,1.37)	1.11 (0.89,1.37)
Black race (vs. other races)		1.03 (0.86,1.22)	1.02 (0.86,1.22)	1.02 (0.86,1.22)	1.02 (0.86,1.22)
Married (vs. Unmarried)		0.79* (0.68,0.92)	0.79 (0.68,0.92)	0.80 (0.69,0.92)	0.80 (0.69,0.92)
Patient is Service Connected (vs. not Connected)		0.98 (0.85,1.13)	0.98 (0.85,1.13)	0.98 (0.85,1.12)	0.98 (0.85,1.12)
Homeless (vs. not Homeless)		1.32* (1.10,1.59)	1.32 (1.10,1.59)	1.32 (1.10,1.59)	1.32 (1.10,1.59)
Serious Mental Illness (vs. not Serious)		1.02 (0.89,1.17)	1.02 (0.89,1.17)	1.02 (0.89,1.17)	1.02 (0.89,1.17)
Last VA use was inpatient (vs. other service)		3.05* (2.24,4.16)	3.04* (2.24,4.14)	3.01* (2.21,4.10)	3.00* (2.20,4.08)
Comorbidity					
	1	0.96 (0.81,1.14)	0.96 (0.81,1.14)	0.96 (0.81,1.13)	0.96 (0.81,1.13)
	2	1.08 (0.91,1.29)	1.08 (0.91,1.30)	1.08 (0.91,1.29)	1.08 (0.91,1.29)
	3 or more	1.43* (1.21,1.70)	1.44* (1.21,1.71)	1.43* (1.21,1.70)	1.43* (1.21,1.70)
Facility is CBOC (vs. not CBOC)		0.67 (0.13,3.56)	0.86 (0.15,5.00)	0.57 (0.10,3.16)	0.65 (0.12,3.60)
Total number of patients for site (median centered)		1.00 (1.00,1.00)	1.00 (1.00,1.00)	1.00 (1.00,1.00)	1.00 (1.00,1.00)
Total number of eligible patients on patient list for site (median centered)		0.99 (0.97,1.01)	0.99 (0.97,1.02)	0.99 (0.97,1.01)	0.99 (0.97,1.01)
Bayesian Information Criterion (BIC)		7241.7	7218.6	7271.8	7727.7

*$p < 0.01$

Table S2: Attempted Contact

Attempted Contact		Entrepreneurial Culture OR (.95 CI)	Hierarchical Culture OR (.95 CI)	Task Climate OR (.95 CI)	Relational Climate OR (.95 CI)
Immediate vs. Delayed EREP		0.41 (0.15,1.17)	0.36 (0.12,1.02)	0.34* (0.12,0.99)	0.36 (0.12,1.03)
Culture/Climate (standardized)		0.56 (0.31,1.02)	0.56 (0.31,1.03)	0.73 (0.41,1.30)	0.71 (0.40,1.25)
Immediate vs. Delayed EREP × Culture/Climate		1.63 (0.70,3.79)	2.13 (0.95,4.80)	1.30 (0.53,3.21)	1.59 (0.66,3.86)
Time					
	6 months (vs. 0 months)	3.71* (2.93,4.69)	3.65* (2.89,4.61)	3.80* (2.99,4.84)	3.71* (2.92,4.71)
	12 months (vs. 0 months)	12.86* (10.16,16.28)	12.70* (10.04,16.07)	12.78* (10.02,16.30)	12.35* (9.70,15.73)
Immediate vs. Delayed EREP × Time					
	6 months (vs. 0 months)	3.38* (2.42,4.74)	3.37* (2.41,4.72)	3.73* (2.64,5.27)	3.72* (2.64,5.25)
	12 months (vs. 0 months)	2.35* (1.67,3.31)	2.44* (1.73,3.44)	2.52* (1.76,3.59)	2.58* (1.81,3.68)
Culture/Climate × Time					
	6 months (vs. 0 months)	1.19 (0.94,1.51)	1.36 (1.06,1.74)	0.89 (0.71,1.13)	0.99 (0.77,1.27)
	12 months (vs. 0 months)	1.15 (0.90,1.45)	1.35 (1.06,1.71)	1.02 (0.81,1.29)	1.15 (0.90,1.47)
Immediate vs. Delayed EREP × Culture/Climate × Time					
	6 months (vs. 0 months)	1.59* (1.12,2.25)	1.15 (0.82,1.63)	1.65* (1.14,2.38)	1.27 (0.88,1.84)
	12 months (vs. 0 months)	0.99 (0.69,1.41)	0.94 (0.67,1.34)	0.98 (0.67,1.43)	0.79 (0.55,1.16)
Baseline Age (centered at 21 years)		0.99* (0.98,0.99)	0.99* (0.98,0.99)	0.99* (0.98,0.99)	0.99* (0.98,0.99)
Male (vs. Female)		0.99 (0.81,1.21)	0.99 (0.81,1.21)	0.99 (0.81,1.21)	0.99 (0.81,1.21)
Black race (vs. other races)		1.03 (0.87,1.23)	1.03 (0.87,1.23)	1.03 (0.87,1.23)	1.03 (0.87,1.22)
Married (vs. Unmarried)		1.04 (0.90,1.20)	1.04 (0.90,1.20)	1.04 (0.90,1.20)	1.04 (0.90,1.20)
Patient is Service Connected (vs. not Connected)		1.00 (0.87,1.15)	1.00 (0.87,1.15)	1.00 (0.87,1.15)	1.00 (0.87,1.15)
Homeless (vs. not Homeless)		0.93 (0.78,1.12)	0.93 (0.78,1.12)	0.93 (0.78,1.12)	0.93 (0.78,1.12)
Serious Mental Illness (vs. not Serious)		0.87 (0.76,1.00)	0.87 (0.76,1.00)	0.87 (0.76,1.00)	0.87 (0.76,1.00)
Last VA use was inpatient (vs. other service)		1.99* (1.48,2.69)	2.00* (1.48,2.69)	1.98* (1.47,2.67)	1.98* (1.47,2.67)
Comorbidity					
	1	0.88 (0.75,1.03)	0.88 (0.75,1.04)	0.88 (0.75,1.03)	0.88 (0.75,1.03)
	2	0.99 (0.83,1.17)	0.99 (0.83,1.17)	0.99 (0.83,1.17)	0.99 (0.83,1.17)
	3 or more	0.88 (0.74,1.04)	0.88 (0.74,1.04)	0.88 (0.74,1.04)	0.88 (0.74,1.04)
Facility is CBOC (vs. not CBOC)		0.43 (0.10,1.80)	0.50 (0.12,2.09)	0.40 (0.09,1.71)	0.42 (0.10,1.80)
Total number of patients for site (median centered)		1.00 (1.00,1.00)	1.00 (1.00,1.00)	1.00 (1.00,1.00)	1.00 (1.00,1.00)
Total number of eligible patients on patient list for site (median centered)		0.99 (0.97,1.01)	1.00 (0.98,1.01)	0.99 (0.97,1.01)	1.00 (0.98,1.02)
Bayesian Information Criterion		7511.1	7524.0	7531.0	7536.5

*$p < 0.01$

Table S3: Completed Contact

Completed Contact	Entrepreneurial Culture OR (.95 CI)	Hierarchical Culture OR (.95 CI)	Task Climate OR (.95 CI)	Relational Climate OR (.95 CI)
Immediate vs. Delayed EREP	1.00 (0.44,2.28)	0.97 (0.43,2.19)	0.91 (0.39,2.14)	0.92 (0.40,2.15)
Culture/Climate (standardized)	0.67 (0.40,1.10)	0.66 (0.40,1.10)	0.96 (0.59,1.57)	0.91 (0.55,1.49)
Immediate vs. Delayed EREP × Culture/Climate	1.31 (0.65,2.64)	1.84 (0.95,3.60)	0.85 (0.39,1.82)	0.98 (0.46,2.08)
Time				
6 months (vs. 0 months)	2.57* (1.76,3.74)	2.54* (1.74,3.70)	2.60* (1.76,3.85)	2.51* (1.71,3.68)
12 months (vs. 0 months)	5.44* (3.82,7.75)	5.43* (3.82,7.73)	5.66* (3.92,8.16)	5.45* (3.81,7.81)
Immediate vs. Delayed EREP × Time				
6 months (vs. 0 months)	1.59 (0.96,2.63)	1.58 (0.95,2.62)	1.64 (0.97,2.79)	1.66 (0.98,2.78)
12 months (vs. 0 months)	1.22 (0.76,1.98)	1.24 (0.76,2.00)	1.15 (0.69,1.90)	1.18 (0.72,1.94)
Culture/Climate × Time				
6 months (vs. 0 months)	1.23 (0.85,1.78)	1.33 (0.91,1.93)	0.91 (0.63,1.32)	1.03 (0.70,1.53)
12 months (vs. 0 months)	1.07 (0.76,1.52)	1.15 (0.81,1.62)	0.83 (0.58,1.17)	0.94 (0.65,1.35)
Immediate vs. Delayed EREP × Culture/Climate × Time				
6 months (vs. 0 months)	1.07 (0.65,1.77)	0.83 (0.52,1.34)	1.29 (0.74,2.23)	1.01 (0.59,1.75)
12 months (vs. 0 months)	0.92 (0.58,1.48)	0.81 (0.52,1.26)	1.11 (0.66,1.88)	0.96 (0.57,1.63)
Baseline Age (centered at 21 years)	1.00 (0.99,1.00)	1.00 (0.99,1.00)	1.00 (0.99,1.00)	1.00 (0.99,1.00)
Male (vs. Female)	0.89 (0.69,1.16)	0.89 (0.69,1.16)	0.89 (0.69,1.15)	0.89 (0.69,1.15)
Black race (vs. other races)	0.96 (0.76,1.22)	0.96 (0.76,1.23)	0.96 (0.76,1.22)	0.96 (0.76,1.22)
Married (vs. Unmarried)	1.41* (1.18,1.68)	1.41* (1.18,1.69)	1.41* (1.18,1.68)	1.41* (1.18,1.68)
Patient is Service Connected (vs. not Connected)	1.29* (1.08,1.54)	1.29* (1.08,1.54)	1.29* (1.08,1.54)	1.29* (1.08,1.54)
Homeless (vs. not Homeless)	0.48* (0.35,0.65)	0.48* (0.35,0.65)	0.48* (0.35,0.65)	0.48* (0.35,0.65)
Serious Mental Illness (vs. not Serious)	0.73* (0.61,0.88)	0.73* (0.61,0.88)	0.73* (0.61,0.88)	0.73* (0.61,0.88)
Last VA use was inpatient (vs. other service)	0.69 (0.43,1.10)	0.69 (0.43,1.10)	0.69 (0.43,1.10)	0.69 (0.43,1.10)
Comorbidity				
1	0.90 (0.73,1.13)	0.91 (0.73,1.13)	0.90 (0.72,1.13)	0.90 (0.72,1.13)
2	1.22 (0.98,1.51)	1.22 (0.98,1.51)	1.22 (0.98,1.51)	1.22 (0.98,1.51)
3 or more	1.00 (0.80,1.26)	1.00 (0.80,1.26)	1.00 (0.79,1.26)	1.00 (0.79,1.26)
Facility is CBOC (vs. not CBOC)	0.61 (0.21,1.78)	0.66 (0.23,1.95)	0.55 (0.18,1.64)	0.57 (0.19,1.70)
Total number of patients for site (median centered)	1.00 (1.00,1.00)	1.00 (1.00,1.00)	1.00 (1.00,1.00)	1.00 (1.00,1.00)
Total number of eligible patients on patient list for site (median centered)	0.99 (0.98,1.01)	0.99 (0.98,1.01)	0.99 (0.98,1.01)	0.99 (0.98,1.01)
Bayesian Information Criterion	4873.4	4875.5	4877.9	4880.8

*$p < 0.01$
